# Differentiated white blood cell count measures as a predictor of weight in anorexia nervosa

**DOI:** 10.1192/bjo.2021.657

**Published:** 2021-06-18

**Authors:** Benjamin Cross, Matthew Cahill

**Affiliations:** 1 East Lancashire Hospitals NHS Trust; 2 Cheshire & Wirral Partnership NHS Foundation Trust

## Abstract

**Aims:**

To explore the relationship between weight, BMI and differentiated white cell count in patients with anorexia nervosa that have commenced refeeding protocols.

Hypotheses:

- 1. Weight and BMI will be positively correlated with differentiated white blood cell measures.
- 2. Regression analyses will indicate that differentiated white blood cell measures can predict weight and BMI.

**Background:**

Anorexia nervosa is a serious mental illness with the highest psychiatric mortality rate. It is characterised by an extreme overvaluation of body image and a preoccupation with weight loss that is achieved through calorie restriction, purging or laxative abuse. The eventual malnutrition that is as a result of self-starvation causes significant negative physical sequelae. One such consequence is the detrimental effect that starvation has on the immune system, leading to bone marrow suppression and cytopenias. In obesity, weight loss has been shown to cause reductions in differentiated white blood cell counts, which are often reduced in anorexia. No studies to date have explored whether weight is related to white blood cell count in anorexia, despite being a common finding in patients.

**Method:**

All subjects were inpatients or ex-inpatients on an eating disorder unit in the Wirral, NW England. Subjects were assessed according to an inclusion criterion. 12 consecutive weekly weights, BMIs and differentiated white blood cell measures were collected for analysis in SPSS. Correlational and regression analyses were then performed to examine the relationship between variables.

**Result:**

302 data points from 26 individuals were used in the final analysis. Weight and BMI both moderately positively correlated with white cell count, r = 0.364 p < 0.001 N = 302 and r = 0.521 p < 0.001 N = 302, respectively. Weight and BMI also moderately positively correlated with all differentiated measures of white blood cell count. Linear regression revealed a significant regression equation between weight, BMI and white blood cell count: (F (1,301) = 22.482, p < 0.001) R2 = 0.69 and (F (1,301) = 112.244 p < 0.001) R2 = 0.272, respectively.

**Conclusion:**

We have demonstrated that there is a moderately positive relationship between weight, BMI and differentiated white blood cell measures. Furthermore, in line with our hypotheses, differentiated white blood cell measures have some power to predict weight and BMI. This further implicates the effect that starvation and anorexia nervosa have on the immune system. It also highlights the possibility that it might be possible to use immunological markers in order to deduce whether weights have been falsified by those with anorexia nervosa.

